# Discrimination of Radix Polygoni Multiflori from different geographical areas by UPLC-QTOF/MS combined with chemometrics

**DOI:** 10.1186/s13020-017-0155-8

**Published:** 2017-12-08

**Authors:** Jin-Fa Tang, Wei-Xia Li, Fan Zhang, Yu-Hui Li, Ying-Jie Cao, Ya Zhao, Xue-Lin Li, Zhi-Jie Ma

**Affiliations:** 1 The First Affiliated Hospital of Henan University of Chinese Medicine, No. 19, Renmin Road, Jinshui District, Zhengzhou, 450000 People’s Republic of China; 2 Beijing Friendship Hospital Affiliated to Capital Medical University, No. 95, Yongan Road, Xuanwu District, Beijing, 100050 China

**Keywords:** UPLC-QTOF/MS, Radix Polygoni Multiflori, Different geographical origins, Chemical markers, Partial least squared discriminant analysis

## Abstract

**Background:**

Nowadays, Radix Polygoni Multiflori (RPM, Heshouwu in Chinese) from different geographical origins were used in clinic. In order to characterize the chemical profiles of different geographical origins of RPM samples, ultra-high performance liquid chromatography quadrupole time of flight mass spectrometry (UPLC-QTOF/MS) combined with chemometrics (partial least squared discriminant analysis, PLS‑DA) method was applied in the present study.

**Methods:**

The chromatography, chemical composition and MS information of RPM samples from 18 geographical origins were acquired and profiled by UPLC-QTOF/MS. The chemical markers contributing the differentiation of RPM samples were observed and characterized by supervised PLS‑DA method of chemometrics.

**Results:**

The chemical composition differences of RPM samples derived from 18 different geographical origins were observed. Nine chemical markers were tentatively identified which could be used as specific chemical markers for the differentiation of geographical RPM samples.

**Conclusions:**

UPLC-QTOF/MS method coupled with chemometrics analysis has potential to be used for discriminating different geographical TCMs. Results will help to develop strategies for conservation and utilization of RPM samples.

**Electronic supplementary material:**

The online version of this article (10.1186/s13020-017-0155-8) contains supplementary material, which is available to authorized users.

## Background

Radix Polygoni Multiflori (RPM, Heshouwu in Chinese) is the dried root tuber of *Polygonum multiflorum* Thunb. (Fam. Polygonaceae). As one of the most popular and precious traditional Chinese medicines (TCMs), it is officially documented in the Chinese Pharmacopoeia for calming the nerves, nourishing blood, activating channels and collaterals, tonifying liver and kidneys, and preventing the premature graying of hair. Many 1000 years of clinical practice of TCM has demonstrated the effect of RPM in terms of preventing dementia and improving memory [1]. As a traditional medicine and dietary supplement for health, it has also been considered effective in antiaging and increasing longevity [2, 3]. According to modern researches, RPM has the pharmacological effects of enhancing immunity, anti-atherosclerosis, anti-inflammatory, antibacterial, anti-cancer, anti-mutagenic, anti-oxidation, increasing DNA repair, and improving adipose metabolism [4–6].

With the extensive application of RPM, its safety has drawn widespread attention. More and more literatures showed that RPM and RPM-containing herbal products had the adverse effects of hepatotoxicity [7]. The RPM dose in the Chinese Pharmacopoeia (2005 edition) is 6–12 g [8]. According to the safety considerations, the recommended dose of RPM was adjusted to 3–6 g in the 2010 edition of the Chinese Pharmacopoeia [9]. In addition, the safety, quality and efficacy of RPM samples may vary greatly because of the different geographical origins. As well known, medicinal herbs in authentic producing areas had the best quality, which can produce the best pharmacological effect. The place where authentic medicinal herbs produced is called the “trueborn area”. RPM is widely distributed in China’s southwest, central, south, east and other regions, including Sichuan, Yunnan, Guizhou, Chongqing, Guangdong, Guangxi, Jiangsu, Anhui, Hubei, Hunan, Henan, Jiangxi, Shanxi, Gansu and other provinces and cities [10]. Owing to its many origins, the “trueborn area” of RPM is still being studied. It is consensus that the effect of Chinese medicine relies on the role of its multi-component. There are large difference among the chemical composition and content of RPMs because of its different species and origins, which will cause a greater impact on its efficacy. Therefore, the distinction among RPM samples from different origins is essential for determining the trueborn area of RPM and for selecting good quality RPM to treat diseases.

The introduction of new analytical techniques and the application of novel data analysis methods have greatly promoted the quality assurance of TCM. From the literatures summary, we found that fingerprinting quality control of RPM from different geographical origins was determined by thin-layer chromatography (TLC) scanning and high-performance liquid chromatography (HPLC) [11, 12]; the quality of various commercial specifications of RPM and its dregs was evaluated by HPLC [13]; the quality control of RPM from different origins was determined by infrared spectrum (IR), inductively coupled plasma-atomic emission spectrometer (ICP-AES) and LC–mass spectrometry (MS), etc. [14–16]. However, the authentic correlation between geographical distribution regions and chemical variation in RPMs has been rarely reported.

It was reported that RPM mainly contains anthraquinones, stilbene glycosides, phospholipids, phenols, flavonoids, etc. [17]. The present study is aimed to classify and characterize RPM samples from different geographical origins based on the chemical compounds by chemometrics. Chemometrics is an interdisciplinary science involving mathematics and statistics, chemistry and computer science. In recent years, chemometrics have gained more attention along with the development of computer science. Chemometrics combined with liquid chromatography and other spectrometric methods are widely used in many fields concerning TCMs, such as the comparison of different species [18], quality control and modernization of TCM [19]. Herein, eighteen RPM samples from 10 counties of 4 provinces were analyzed using ultra-high performance liquid chromatography quadrupole time of flight mass spectrometry (UPLC-QTOF/MS). And partial least squared discriminant analysis (PLS‑DA) of chemometrics approach was applied to classify different RPM samples and find chemical variables that contribute to the differentiation of RPMs. Furthermore, the Progenesis QI software (v2.0, Waters Corporation, Milford, USA) with fast, objective, and reliable characteristics was used for the chemometrics statistical analysis, which had been already used to found the chemical differences among the different extracts of RPM and RPM Praeparata [20], but it only analyzed the differences between the water and ethanol extracts of RPM and RPM Praeparata. Therefore, it would also be feasible to use this method to find different chemical markers among RPMs from different geographical origins. The results can provide more effective strategy guidance for the utilization and domestication of RPM.

## Methods

The Minimum Standards of Reporting Checklist (Additional file 1) contains details of the experimental design, and statistics, and resources used in this study.

### Chemicals and reagents

Acetonitrile (HPLC grade) and formic acid were purchased from Merck KGaA (Darmstadt, Germany); ultra-pure water was purified by a Milli-Q system (Milford, MA, USA).

### Plant materials

Seventeen species of planted or wild RPM samples were collected from 10 counties, 4 provinces of China; and one kind of RPM sample (S13) was purchased from pharmacies (Table 1). All the herbal samples were authenticated by the authors. The corresponding voucher specimens were stored in the laboratory for drug metabolism and pharmacokinetics (DMPK) Research of Herbal Medicines, the First Affiliated Hospital of Henan University of Chinese Medicine.

**Table 1:** Geographical information of 18 RPM samples

No.	Source	Longitude	Latitude
S1	Cultivated in Xinzhou town, Huangping county, Guizhou province	107.92213	26.98450
S2	Wild, Xinzhou town, Huangping county, Guizhou province	107.92213	26.99918
S3	Wangsi town, Duyun county, Guizhou province	107.51576	26.30596
S4	Huaxi district, Guiyang city, Guizhou province	107.27755	26.73151
S5	Cultivated in Meitan county, Zunyi city, Guizhou province for 2 years	107.45728	28.02350
S6	Cultivated in Meitan county, Zunyi city, Guizhou province for 3 years	107.45728	28.02350
S7	Guizhou Academy of Agricultural Sciences (Huaxi district, Guiyang city, Guizhou province)	107.27755	26.73151
S8	Baiduo village, Shibing county, Guizhou province for 2 years	108.15923	27.14368
S9	Baiduo village, Shibing county, Guizhou province for 3 years	108.15923	27.14368
S10	Niudachang town, Shibing county, Guizhou province for 2 years	107.92594	27.14054
S11	Niudachang town, Shibing county, Guizhou province for 3 years	107.92594	27.14054
S12	Wild, Shibing county, Guizhou province	108.06787	27.12923
S13	Changhao Chinese Medicine Development Co., Ltd. (Shibing county, Guizhou province)	107.94637	26.55361
S14	Zhenjiang town, Gaozhou city, Guangdong province	110.84849	21.94484
S15	Shigu town, Gaozhou city, Guangdong province	110.86943	21.95980
S16	Yangchun city, Guangdong province	111.79154	22.17044
S17	Luanchuan county, Luoyang city, Henan province	111.61577	33.78570
S18	Panzhihua city, Miyi county, Sichuan province	102.11034	26.89069

### Sample preparation

The 18 RPM samples were sliced, dried, and powdered. The powdered samples were screened trough no. 4 sieve, respectively; and 0.25 g was extracted with 25 mL 70% ethanol for 30 min by reflux extraction method, cooled at room temperature and weighted. The reduced weight was complemented by 70% ethanol and mixed well. After standing, the supernatant was filtered through filter paper. During the process, 18 RPM samples were prepared 3 replicates. Before UPLC-QTOF/MS analysis, the filtered supernatant was filtered through a 0.22 μm microporous membrane and 2 μL aliquot was injected.

In addition, quality control (QC) sample was prepared by mixing 100 μL supernatants of 18 geographical RPM samples to validate stability of LC–MS system. It was injected for 3 times before beginning the whole sample list to condition or balance the system. During the analytical run, QC sample was injected every 9 RPM samples to further monitor and investigate the stability and analytical variability of the system. After that, the change degree of the analytical system in the analysis process could be obtained and determined, which was critical for assessing the variation and reliability of the analytical results.

### UPLC-QTOF/MS conditions

Samples were analyzed using a Waters ACQUITY UPLC I-Class system (Waters Corporation, Milford, USA). An Acquity UPLC HSS T3 C18 column (2.1 mm i.d. × 100 mm, 1.8 μm) was used for chromatographic separation. All samples were run in a random and non-grouped order. The flow phases consisted of 0.1% formic acid in water (A) and acetonitrile (B). The program of gradient elution was set as follows: 0−16 min, 5−60% B; 16−20 min, 60−100% B. The flow rate was 0.4 mL/min. The temperatures of column oven and auto-sampler were maintained at 35 and 10 °C during the analysis, respectively. The sample injection volume was 2 μL.

MS spectrometry detection was operated on a Waters Xevo G2-XS QTOF/MS (Waters, Manchester, UK) equipped with the UPLC system through an electrospray ionization (ESI) interface in negative and positive ion modes. The ESI source parameters were maintained as follows: capillary voltage 1.0 kV, cone voltage 40 V, source temperature 110 °C, desolvation temperature 450 °C. Nitrogen was used as cone gas and desolvation gas with flow of 50 and 800 L/h, respectively. Argon was used as collision gases. The acquisition range of MS scanning was from *m*/*z* 50 to 1200 Da in MS^E^ continuum mode. By using a collision energy ramp from 10 to 30 V, the MS/MS fragment information was obtained. The mass accuracy and reproducibility of UPLC-QTOF/MS was validated by the reference lock mass of leucine-enkephalin (ESI^+^: *m*/*z* 556.2771; ESI^−^: *m*/*z* 554.2615) with the concentration of 100 pg/μL and the flow rate of 10 μL/min. Data acquisition was performed using Masslynx™ v 4.1 (Waters, Manchester, UK).

### Data processing

All raw data of RPM samples in the LC–MS runs were loaded on Progenesis QI software (v2.0). By using the “assess all runs in the experiment for suitability”, QC2 was automatically selected as the alignment reference. Next, the peaks of all other runs were aligned by comparison with QC2. After that, the experiment design (QC group and S1–S18 groups) was set, the peaks of all samples were picked and convoluted. And then, all data of the peaks were exported into the EZinfo software (v3.0) for PLS‑DA analysis. The necessary data were filtered and then were imported into Progenesis QI software (v2.0) to identify the compounds by its powerful Metascope search engine in the software according to the accurate mass, isotope distribution, fragment ions, collision cross-sectional area and many other parameters.

The significant differences of the markers in different RPM species were analyzed by one-way analysis of variance (ANOVA). The results are shown as mean ± SD. The differences were considered statistically significant at *P* ≤ 0.05.

## Results

### Data analysis by Progenesis QI

**Fig. 1:**
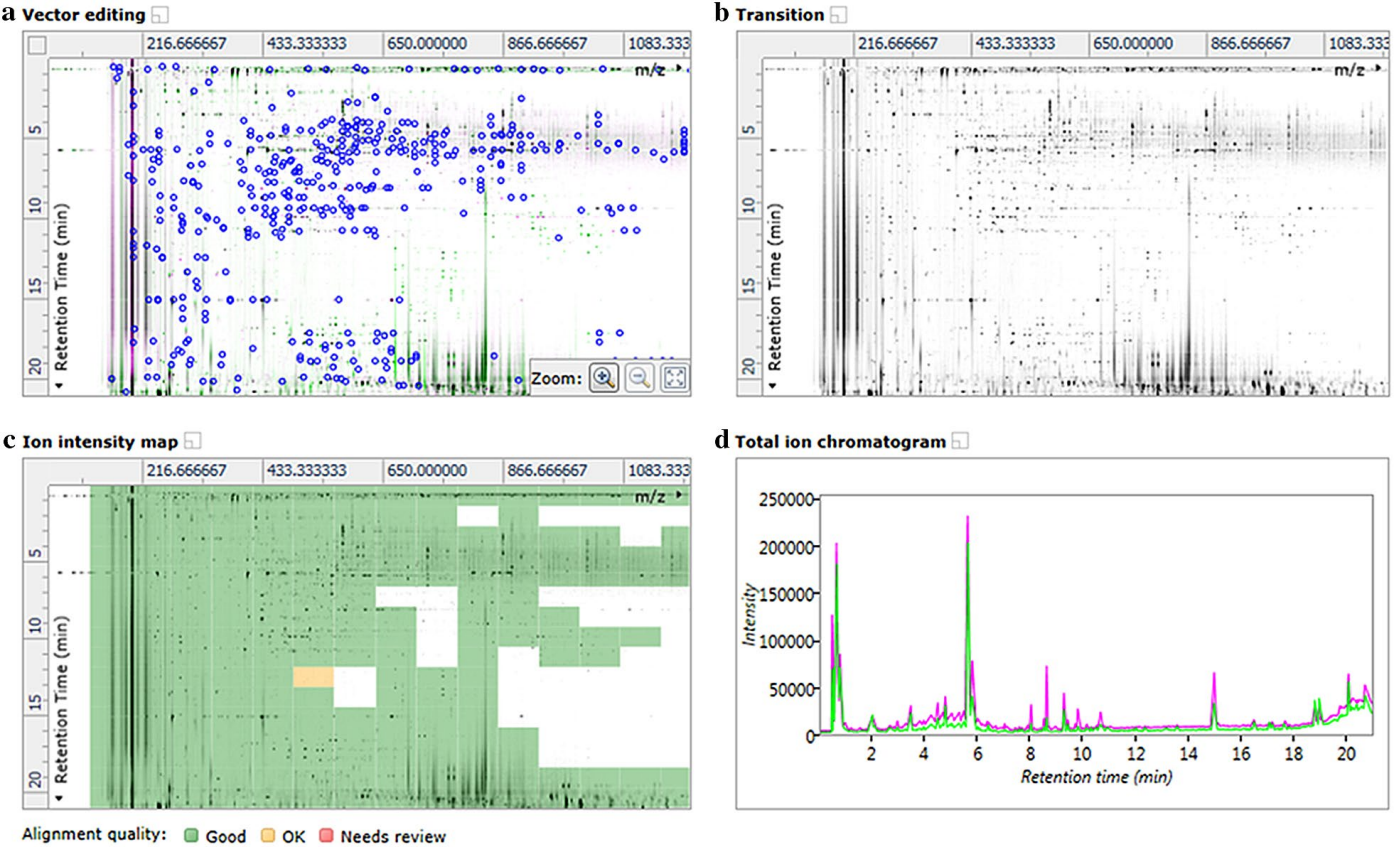
The translation process by Progenesis QI software (**a** vector alignment window; **b** and **c** 2D ion intensity maps; **d** total ion chromatograms, green: QC2; purple: S9b)

Multivariate statistical tools was used to observe all differences among the RPM samples from different geographical origins. Firstly, the 3D LC/MS data acquired by Masslynx™ v 4.1 were converted into a 2D ion intensity map as an exact mass retention time (EMRT) pair by using Progenesis QI. During the process, the RPM QC2 sample was automatically selected as the alignment reference by Progenesis QI, and all other RPM samples were aligned with QC2 as the reference. The representative peak alignment results and chromatograms between QC2 and S14b were shown in Fig. 1. Figure 1a was a vector alignment window, there were 414 vectors; Fig. 1b, c both were 2D ion intensity map. Figure 1c also showed the matching results for peak alignment between QC2 and S9b, the score was 96.1%. The matching score range between QC2 and other RPM sample was from 90.3% (S15c) to 97.9% (S18b). The ordinate of Fig. 1a–c represented the retention time (Rt), and the abscissa of them was *m*/*z*. Figure 1d was the total ion chromatograms (TIC), green and purple chromatograms represented QC2 and S9b, representatively.

Then, the experiment was designed, 60 samples were divided into 19 groups, including QC and S1–S18 groups. Next, all peaks of RPM samples were picked and convoluted. The parameters of sensitivity value was set at 3 and the minimum peak width was set at 0.15 min, respectively. Under the condition, the best balance could be obtained with the most true feature ion signals and the least random noise. Total 24,530 peaks were observed in the 2D ion intensity map, which was shown in Additional file 2: Figure S1. The normalization graphs for the RPM samples are shown in Additional file 3: Figure S2.

### PLS-DA analysis for RPM samples

**Fig. 2:**
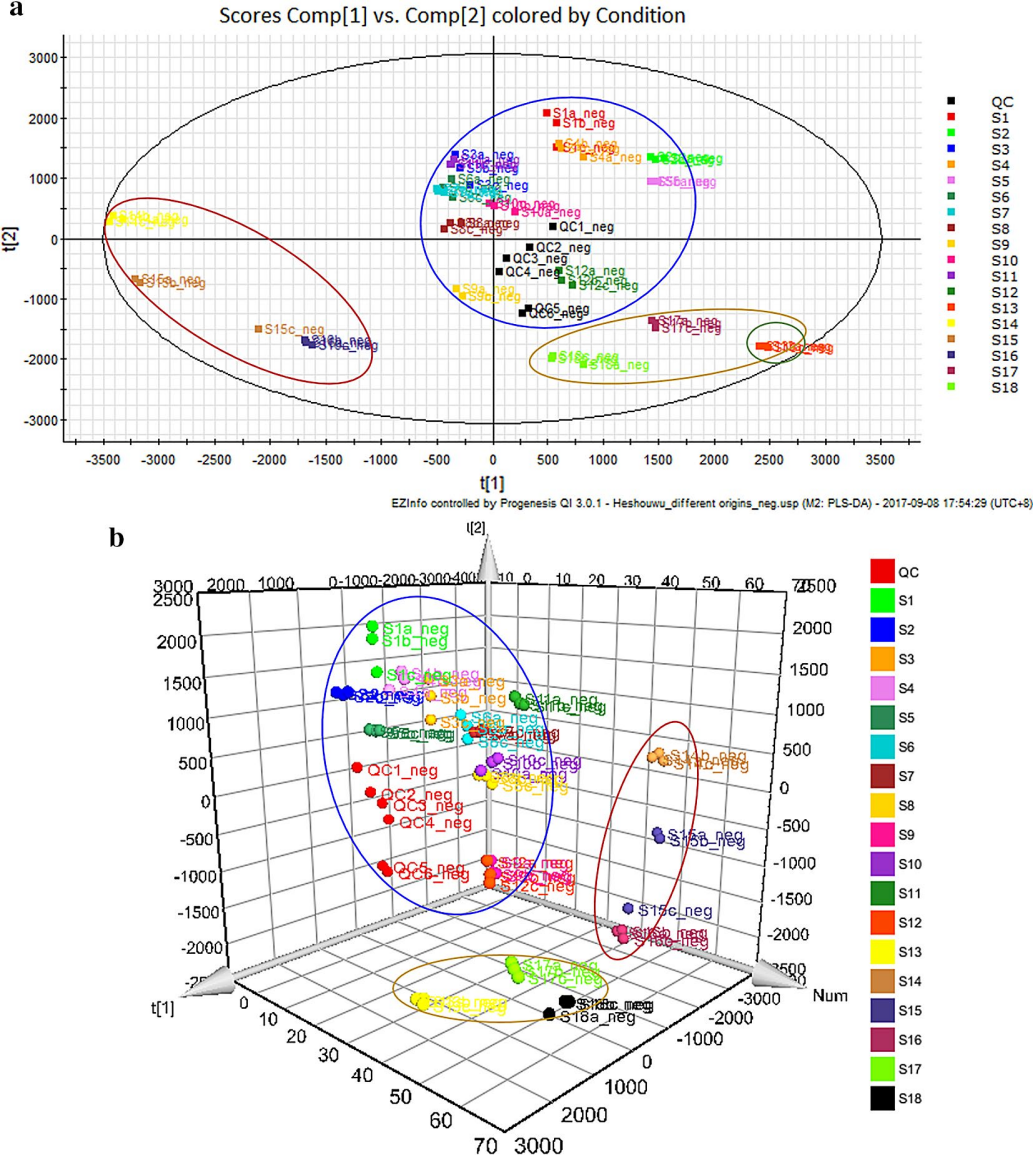
The PLS-DA 2D score plot (**a**) and 3-D score plot (**b**) of RPM samples from different geographical origins

After that, all data were exported into the EZinfo software (v3.0) for PLS-DA analysis. The outliers and classification trends among the 18 kinds of RPM samples could be observed in PLS-DA results (Fig. 2). In the score plot obtained by PLS-DA, there was a clear differentiation between RPM S1–S12 groups and S13 group, indicating that RPM sample from Changhao Chinese Medicine Development Co., Ltd. was very different from other samples in Guizhou province. RPM samples from Guangdong province (S14–S16 groups) clustered together and separated from Guizhou samples. RPM samples from Henan (S17 group) and Sichuan (S18 group) provinces were closer to Guizhou samples, and located father from Guangdong samples. *R* ^2^ *Y* and *Q* ^2^ of the PLS-DA model were 0.771 and 0.634, respectively, which suggested that the PLS-DA model had good adaptability and predictability. Among the RPM samples from the same place of Guizhou province, the samples between S1 and S2 clustered together respectively, indicating that cultivated and wild RPM samples in Xinzhou town had significant difference; the separation between S5 and S6, S8 and S9, S10 and S11 indicated that cultivated RPM samples for 2 and 3 years in Meitan county, Baiduo village, and Niudachang town also had significant difference.

### Identification of chemical markers

**Fig. 3:**
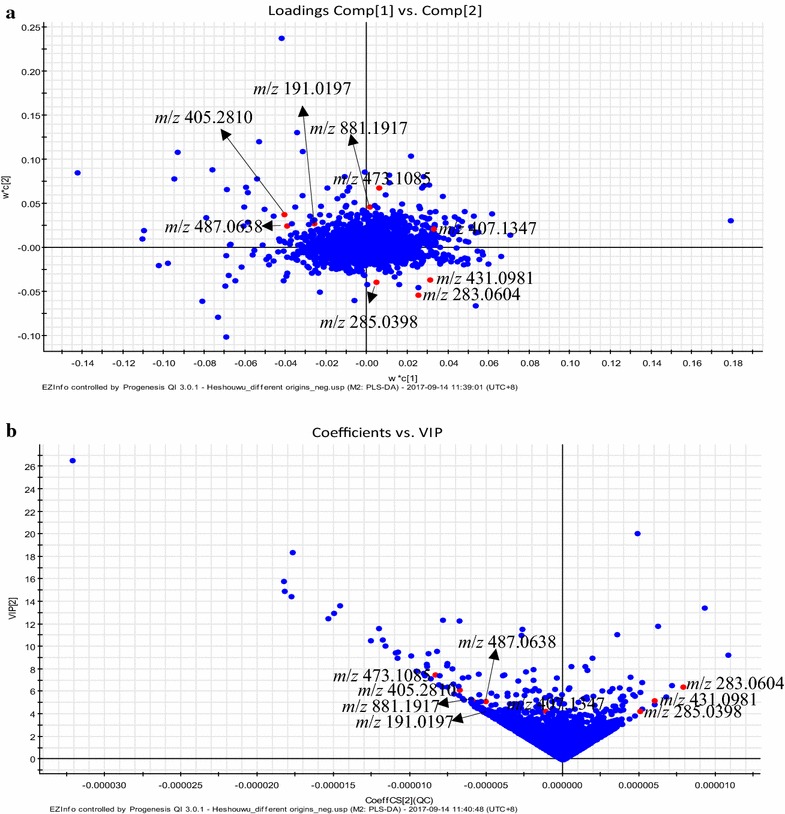
The PLS-DA loading plot (**a**) and VIP plot (**b**) of RPM samples from different geographical origins

Identification of potential chemical markers in RPM samples from different geographical origins was carried out on basis of the retention behavior and mass assignment using Progenesis QI software. First, PLS-DA model was constructed from the EZinfo software. From loading plots (Fig. 3a) and VIP plots (Fig. 3b) of that model, the interested potential biomarkers could be extracted. Additionally, an ANOVA P ≤ 0.05, a maximum fold change ≥ 2 and VIP value > 1 were set as the restriction conditions to select the significant changing compounds and reduce the “false discovery rate (FDR)”. Next, the Progenesis MetaScope, ChemSpider (http://www.chemspider.com/) and Element composition methods of Progenesis QI software was used for preliminary determination of the chemical markers. The mass tolerance between the measured *m*/*z* values and exact mass of the interest compounds, and the relative mass error of the performed theoretical fragmentation both were set to within 5 ppm. Then, under the targeted MS/MS mode, the MS/MS spectrum of chemical markers was obtained. Finally, some chemical markers were identified by comparison with the standard reference; and others were identified by MS/MS spectrum, online database, element composition results, and literatures.

**Fig. 4:**
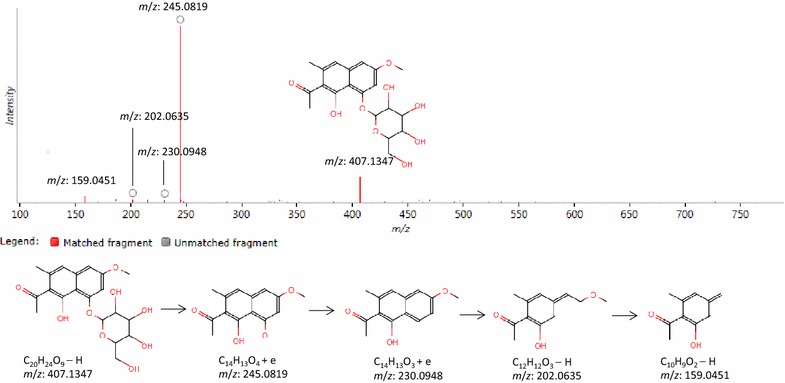
The fragment information of torachrysone-8-*O*-glucoside

According to the protocol detailed above, 9 chemical markers (**C1**–**C9**) in RPM samples from different geographical origins were identified (Table 2). Among them, 4 chemical markers including **C1**, **C3**, **C4** and **C9** were identified by comparing with their reference compounds. Other 5 compounds were tentatively identified on basis of their molecular ion information and fragments generated by precursor ions. Herein, the **C5** with Rt-*m*/*z* of 8.53–407.1347 in negative ion mode was detailed as an example to illustrate the identification process. Firstly, the accurate mass of the marker ([M−H]^−^ at *m*/*z* 407.1347) was found from the mass spectrum (Fig. 4). Secondly, specific MS/MS information about fragmentation pattern of the marker was acquired from QTOF system. The main fragment ions of the marker in the negative ion spectrum were observed at *m*/*z* 245.0819, 230.0948, 202.0635, and 159.0451, which could be the [M−H]^−^ of lost –C_6_H_12_O_5_, –C_6_H_12_O_6_, –C_8_H_14_O_5_, –C_10_H_19_O_6_, respectively. C_20_H_24_O_9_ was located as the candidate due to its high mass accuracy among the possible compounds. Finally, the chemical compound was identified as torachrysone-8-*O*-glucoside (**C5**) according to the ChemSpider database and literature [20, 21].

**Table 2:** Chemical markers in RPM samples from different geographical origins by UPLC-QTOF/MS

No.	*t* (min)	*m*/*z* (−)	Fragment information	Compound name	Compound type	Formula	Max fold change	VIP value
**C1**	0.79	191.0197	191.0197, 173.0091	Citric acid	Organic acids	C_6_H_8_O_7_	4.72	4.11
**C2**	2.86	881.1917	881.1917, 863.1829, 591.1144, 217.0506, 152.0015	3,3′-di-*O*-Galloyl-procyanidin B2	Polyphenols	C_44_H_34_O_20_	8.12	5.12
**C3**	5.68	405.2810	405.2810, 243.1911, 227.0425, 215.0702, 201.0545, 173.0596	2,3,5,4′-Tetrahydroxystilbene-2-*O*-glucoside	Stilbene glycosides	C_20_H_22_O_9_	2.01	6.15
**C4**	8.06	431.0981	431.0981, 269.0455, 265.0506, 263.0350, 253.0506, 241.0506, 227.0350, 224.0478, 210.0322, 182.0373	Emodin-8-*O*-glucoside	Anthraquinone glycosides	C_21_H_20_O_10_	31.99	5.20
**C5**	8.53	407.1347	407.1347, 245.0819, 230.0948, 202.0635, 159.0451	Torachrysone-8-*O*-glucoside	Anthraquinone glycosides	C_20_H_24_O_9_	7.78	4.28
**C6**	8.65	473.1085	473.1085, 456.1062, 431.0983, 269.0455, 265.0506, 253.0506, 240.0428, 227.0350, 224.0478, 210.0322, 182.0373	Emodin-*O*-(acetyl)-hexoside	Anthraquinone derivatives	C_23_H_22_O_11_	4.42	7.54
**C7**	9.30	487.0638	487.0638, 470.0734, 445.0369, 283.0604, 240.0428, 212.0478, 197.0244, 184.0505, 169.0278	Physcion-*O*-(acetyl)-hexoside	Anthraquinone derivatives	C_23_H_22_O_12_	4.73	3.80
**C8**	10.18	285.0398	285.0398, 269.0455, 253.0506, 240.0428, 227.0350, 224.0478, 210.0322, 182.0373	Citreorosein	Anthraquinones	C_15_H_10_O_6_	34.91	4.25
**C9**	12.50	283.0604	283.0604, 240.0428, 212.0478, 197.0244, 184.0505, 169.0278	Physcion	Anthraquinones	C_16_H_12_O_5_	2567.78	6.46

The data of 6 replicates of QC sample were analyzed to evaluate the repeatability of LC–MS method. The relative standard deviations (RSD%) of peak areas, Rt and *m*/*z* were 5.73–13.42, 0–0.25 and 0.00012–0.00301%, respectively. QC sample maintained in auto sampler at 4 °C for 4, 8, 12, 24, 28, 32 h were tested to assess the post-preparation stability of samples. The relative errors of peak areas were < 13.42% demonstrating good repeatability and stability of the method.

**Fig. 5:**
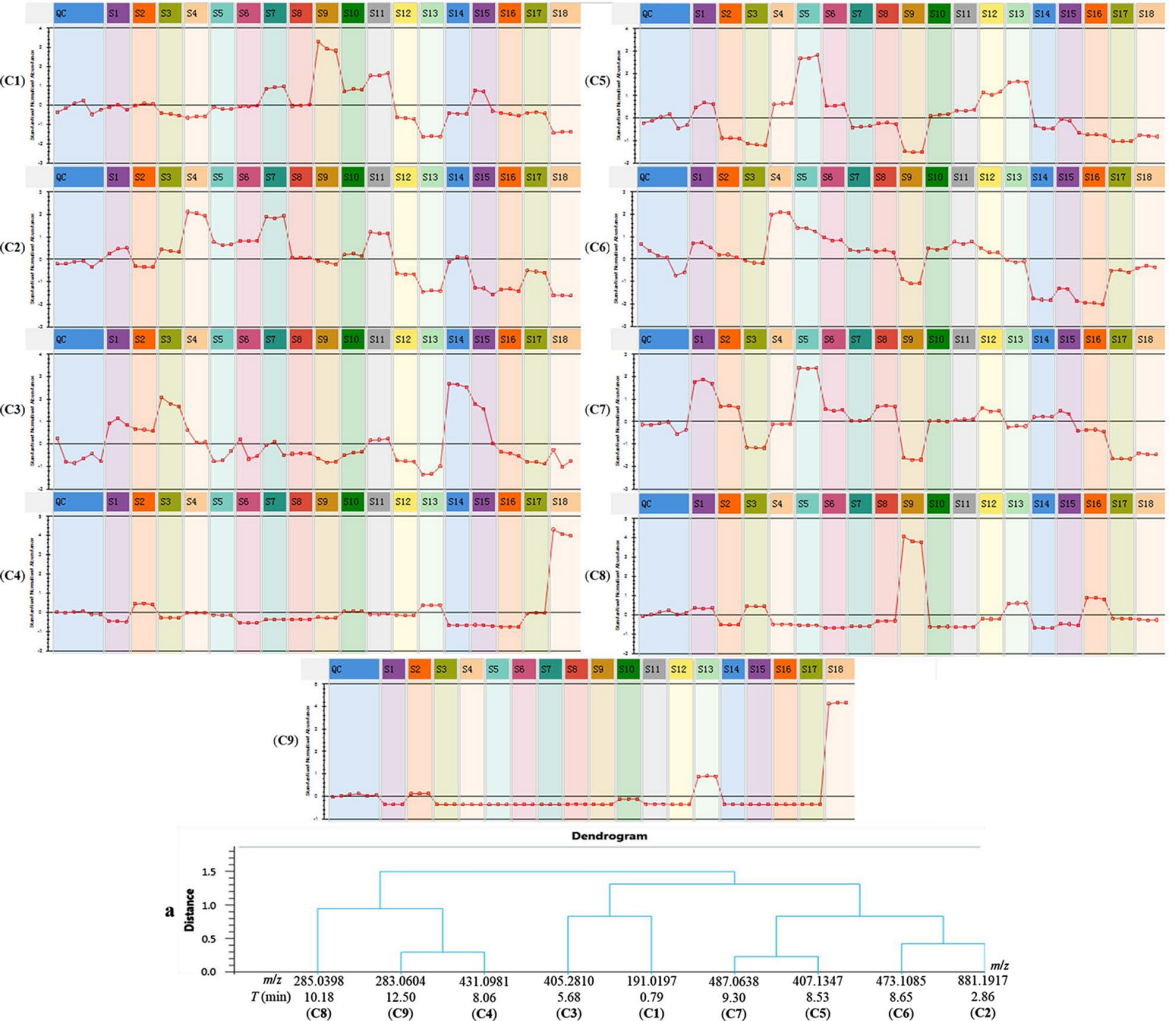
The standard normalized abundance of 9 chemical markers (**C1**–**C9**) and the dendrogram (**a**) of 9 chemical markers

Furthermore, in order to characterize the differences more clearly, the relative intensity of chemical markers in RPM samples from different geographical origins was shown in Table 3 and Fig. 5. The max fold change of **C1**–**C9** were 4.72, 8.12, 2.01, 31.99, 7.78, 4.42, 4.73, 34.91 and 2567.78, respectively. And the results of Fig. 5 **C9** and Table 3 showed that the content of **C9** in 18 RPM samples had the most different, and the content of **C9** in S18 was the highest, which was about 4 time than S13; the content of **C9** in S2 and S10 were similar, which was lower than S13 and S18, but higher than other 15 RPM samples; the content of **C9** in S1, S3–S9, S11, S12, S14–S17 were similar. The content trend of **C4** (Fig. 5 **C4**) in 18 RPM samples was similar with **C9**. The content of **C8** (Fig. 5 **C8**) in S9 was the highest; the content of S1, S3, S13, and S16 were lower than S9, but higher than S2, S4–S8, S10–S12, S14, S15, S17 and S18. The content of S4 and S7 was similar, which was higher than other 16 RPM samples. The content of **C5** (Fig. 5 **C5**) and **C7** (Fig. 5 **C7**) in S5 was the highest, the content of them in S9 was the lowest, but the content of **C7** in S9 was similar with that in S9. The content of **C1** (Fig. 5 **C1**) and **C3** (Fig. 5 **C3**) in S9 and S14 were the highest, respectively; but the content of them in S13 both were the lowest. The content of **C2** (Fig. 5 **C2**) and **C6** (Fig. 5 **C6**) in S4 were the highest, but the content of **C2** in S7 was similar with S4; the content of them in S16 both were the lowest, but the content of **C2** (Fig. 5 **C2**) in S13 and S15 was almost equal to that of S16; and the content of **C6** (Fig. 5 **C6**) in S14 was almost equal to the content in S16.

**Table 3:** The relative intensity of the chemical markers in RPM samples from different geographical origins

Chemical markers	C1	C2	C3	C4	C5	C6	C7	C8	C9
S1	51,705.58 ± 2584.63	11,951.15 ± 645.72	41,725.54 ± 1340.02	6190.75 ± 327.08	38,687.07 ± 1649.29	12,771.68 ± 387.55	93,588.08 ± 1845.63	21,294.27 ± 376.74	200.19 ± 12.50
S2	54,477.74 ± 917.00	8448.42 ± 87.23	38,606.33 ± 469.16	18,825.53 ± 301.36	17,532.28 ± 153.38	11,199.92 ± 238.20	70,742.37 ± 835.72	5498.40 ± 81.59	9020.02 ± 164.72
S3	44,807.13 ± 1130.32	11,820.68 ± 330.10	49,625.66 ± 1886.30	8764.83 ± 106.85	13,463.15 ± 581.06	10,266.89 ± 220.35	32,995.56 ± 377.37	23,010.73 ± 197.83	135.31 ± 6.01
S4	42,325.49 ± 814.97	19,590.50 ± 412.11	35,216.92 ± 2860.68	12,300.10 ± 71.24	39,277.20 ± 327.26	17,174.93 ± 181.83	54,798.00 ± 125.03	6112.91 ± 18.00	32.25 ± 3.28
S5	50,502.99 ± 1160.87	13,260.65 ± 337.26	27,497.91 ± 2333.92	10,675.71 ± 108.05	68,958.14 ± 1118.64	14,959.91 ± 269.53	105,951.47 ± 340.46	5149.43 ± 86.72	142.77 ± 5.33
S6	52,510.17 ± 469.61	13,909.26 ± 26.51	29,965.99 ± 4288.98	5033.78 ± 50.00	38,243.35 ± 601.64	13,510.20 ± 253.37	67,729.01 ± 989.02	2754.53 ± 78.86	112.64 ± 5.46
S7	70,993.90 ± 1120.09	18,915.88 ± 265.25	31,622.93 ± 2802.44	7517.20 ± 59.55	24,700.24 ± 307.49	11,980.72 ± 136.83	57,924.29 ± 528.54	4078.03 ± 49.53	36.85 ± 3.83
S8	53,605.42 ± 785.10	10,332.18 ± 26.08	29,137.10 ± 142.32	7607.70 ± 36.09	26,767.93 ± 389.67	11,810.46 ± 169.16	71,004.30 ± 686.40	9197.88 ± 145.75	419.49 ± 18.62
S9	110,338.57 ± 4435.84	9357.71 ± 360.23	26,242.61 ± 904.28	8703.56 ± 334.27	8860.43 ± 215.65	7481.79 ± 311.97	22,402.21 ± 930.10	85,213.75 ± 2949.23	207.27 ± 6.94
S10	68,329.09 ± 1392.30	10,975.66 ± 244.40	29,228.39 ± 646.70	13,375.96 ± 68.22	32,143.18 ± 499.74	12,191.99 ± 118.92	57,392.75 ± 257.02	3760.47 ± 56.91	4594.62 ± 33.44
S11	83,294.77 ± 1355.53	15,550.55 ± 165.35	34,725.41± 338.49	11,433.86± 102.78	35,063.82 ± 417.58	13,051.54± 162.43	58,825.87 ± 474.70	3568.84 ± 103.00	580.74 ± 14.62
S12	41,004.17 ± 1032.78	6952.98 ± 101.11	26,092.98 ± 252.66	10,560.47 ± 208.07	46,059.83 ± 1052.28	11,851.39 ± 375.25	67,597.12 ± 1589.29	11,122.57 ± 179.55	162.41 ± 4.12
S13	23,363.99 ± 409.93	3334.93 ± 112.08	21,933.99 ± 1748.61	17,956.26 ± 40.22	53,097.08 ± 468.53	10,430.68 ± 150.77	52,453.40 ± 504.74	25,826.56 ± 338.01	23,061.01 ± 317.64
S14	45,590.58 ± 569.55	10,154.77 ± 584.16	56,602.21 ± 661.72	3363.27 ± 49.48	24,145.97 ± 989.02	5032.57 ± 106.25	61,403.82 ± 355.13	2559.13 ± 33.60	319.72 ± 8.80
S15	61,091.53 ± 11,395.42	3528.03 ± 802.85	43,153.78 ± 8584.56	3198.19 ± 365.14	26,207.57 ± 4683.80	5990.59 ± 988.60	59,731.13 ± 9985.09	5800.22 ± 859.43	92.49 ± 7.94
S16	44,684.42 ± 1532.64	3634.70 ± 225.88	29,010.21 ± 859.34	2200.38 ± 79.65	19,619.97 ± 320.79	4492.36 ± 130.95	48,868.69 ± 1139.51	30,643.84 ± 717.00	119.19 ± 4.10
S17	46,299.52 ± 753.18	7457.72 ± 215.09	25,504.16 ± 476.59	12,099.00 ± 86.28	15,555.46 ± 36.35	9026.73 ± 156.75	22,756.80 ± 104.18	11,503.65 ± 83.20	276.60 ± 4.81
S18	27,660.30 ± 536.51	2413.59 ± 48.12	26,851.27 ± 3378.46	70,400.91 ± 2306.84	19,093.18 ± 461.26	9595.14 ± 175.49	27,212.01 ± 692.91	10,298.36 ± 312.60	82,806.08 ± 486.14
QC	50,895.72 ± 5125.28	9259.56 ± 530.38	28,056.00 ± 3725.38	12,474.95 ± 914.73	28,101.47 ± 3305.72	10,350.10 ± 1388.66	52,497.63 ± 4122.08	16,243.25 ± 1997.41	7632.61 ± 928.12

## Discussion

Because of the clinical benefits, TCM is becoming more and more attractive around the world. Therefore, it is an important issue to carry out the quality control study of TCM for its application and development. To control the quality of Chinese medicine and its products, studying the source of TCMs is a key. In the present study, 18 RPM samples were collected from 10 counties, 4 provinces of China. And 9 representative chemical markers related to the differences among the 18 RPM samples were identified. The content of **C1** and **C8** in S9 were the highest; while the content of **C5** and **C7** in S9 were the lowest. The content of **C1**, **C2** and **C3** in S13 and S18 were the lowest. The content of **C2** and **C6** were the highest. The content of **C3** in S14 was the highest. The content of **C4** and **C9** in S18 were the highest. The content of **C5** and **C7** in S5 were the highest. The results suggested that **C1**, **C5** and **C7** could be used as specific chemical markers for S9 and S5; **C1**, **C2** and **C3** could be used as special chemical markers for S13; **C1**, **C2**, **C3**, **C4** and **C9** could be used as specific chemical markers for S18; **C3** could be used as unique chemical markers for S14. The PLS-DA results of different RPM samples showed that the RPM from Guizhou provinces were different from the RPMs of Guangdong, Henan and Sichuan provinces, indicating that RPM samples from Guizhou province had some similarity. The samples from Guangdong, Henan and Sichuan provinces were also clustered together, respectively. And there were significant difference between the RPM sample for 2 and 3 years.

RPM contains a variety of anthraquinones, stilbene glycosides, phospholipids, phenols, flavonoids and tannins. Stilbene glycosides are a class of natural ingredients with a variety of physiological activities. The most active components including aloe-emodine, emodin, rhein and physcione were identified as the antioxidant anthraquinones [22]. The anthraquinone glycoside from RPM could significantly accelerate T and B lymphocytes proliferation in vitro, improve macrophages phagocytosis, increase TNF secretion activity and activity of NK cells, accelerate mixed lymphocyte reaction, and antagonize restraining effect of lymphocyte proliferation by mitomycin [23].

RPM samples from 18 different geographical origins were discriminated using UPLC-QTOF/MS coupled with chemometrics method in the present study. Those chemical markers with significant pharmacological activities could be used to distinguish the geographical origins of RPM samples. The results will help to develop strategies for protection and utilization of RPM samples. Chemometrics technique has potential to be used for discovering active components and evaluating the therapeutic effect and toxicity of TCMs, related to the complex composition and different growth geographical environment, and help us to find the natural substitute for the geographical origins herb medicine depleting more rapidly.

## Conclusion

Classification and distinction of TCMs based on geographical origins are important basis for ensuring their quality and safety. The chemical constitution of RMP samples from different areas were characterized by UPLC-QTOF/MS method. PLS-DA method was applied to classify the samples from different origins. And the marker compounds contributing to the differentiation of RPMs were observed and identified. Nine chemical markers were tentatively identified and semi-quantified in different geographical RPM samples. The individual peak data of those chemical markers with significant content difference in different RPM regions were calculated. Those chemical markers could be applied to distinguish the geographical origins of different geographical RPM samples. The results showed that chemometric technique has potential to be used for discovering active components of TCMs, related to the complex composition and different growth geographical environment, and help us to find the natural substitute for the geographical origins herb medicine depleting more rapidly.

## Additional files

**Additional file 1.** The Minimum Standards of Reporting Checklist.

**Additional file 2: Figure S1.** The peak picking-ion map.

**Additional file 3: Figure S2.** The normalization graphs of RPM samples from different geographical origins.

## Abbreviations

RPM: Radix Polygoni Multiflori
UPLC-QTOF/MS: ultra-high performance liquid chromatography quadrupole time of flight mass spectrometry
PLS‑DA: partial least squared discriminant analysis
TCM: traditional Chinese medicines
TLC: thin-layer chromatography
HPLC: high-performance liquid chromatography
IR: infrared spectrum
ICP-AES: inductively coupled plasma-atomic emission spectrometer
MS: mass spectrometry
DMPK: drug metabolism and pharmacokinetics
QC: quality control
EMRT: exact mass retention time
TIC: total ion chromatograms
ANOVA: analysis of variance
FDR: false discovery rate
