# Supplementary figures and images for: Discrimination of Radix Polygoni Multiflori from different geographical areas by UPLC-QTOF/MS combined with chemometrics
Source: Chin Med. 2017 Dec 8;12:34. doi: 10.1186/s13020-017-0155-8 (PMC5721361; doi:10.1186/s13020-017-0155-8)

## Slide 1
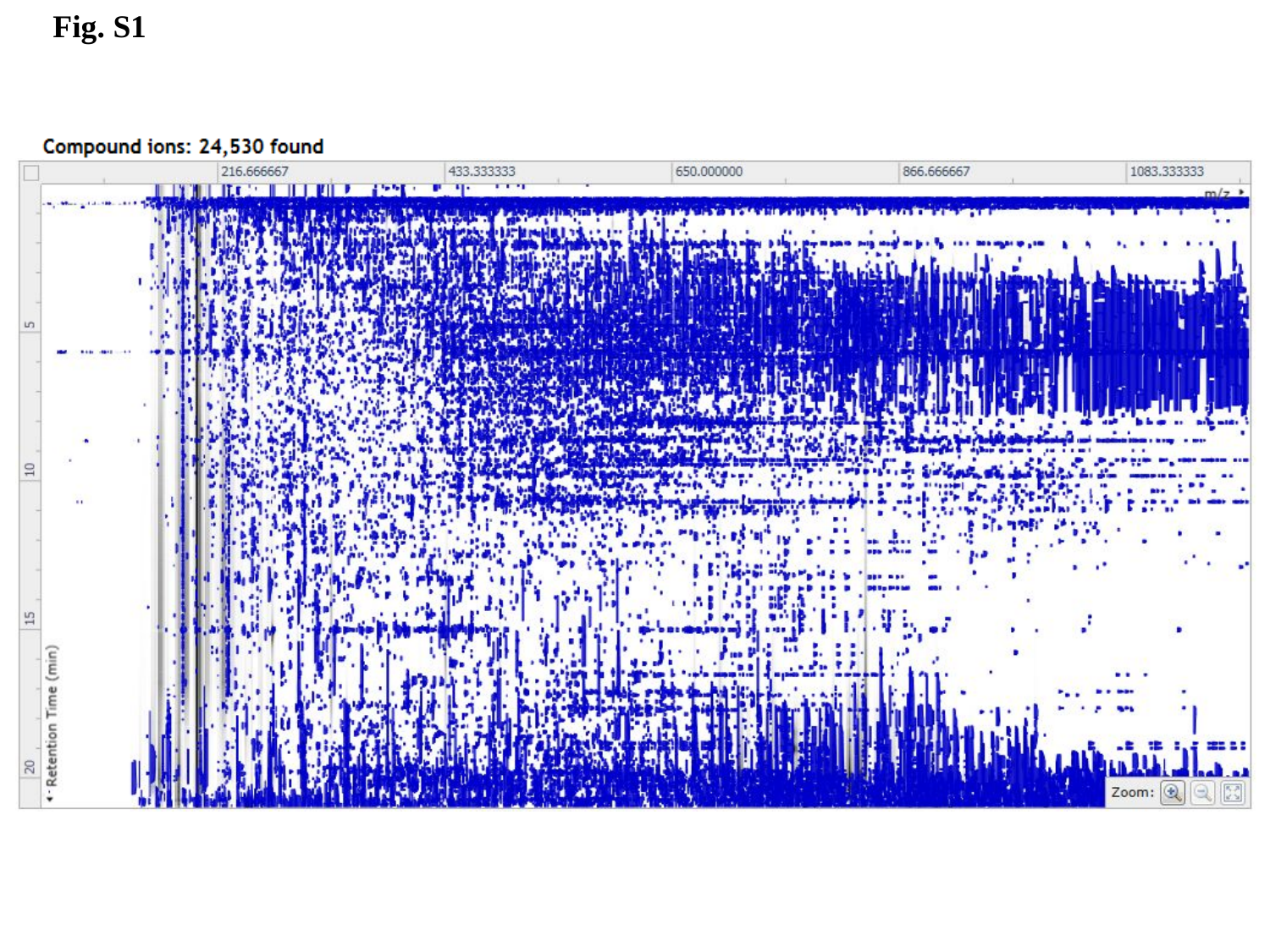

Fig. S1

Supplement: Supplementary file 2 — Additional file 2: Figure S1. The peak picking-ion map. [file 13020_2017_155_MOESM2_ESM.pptx]

## Slide 1
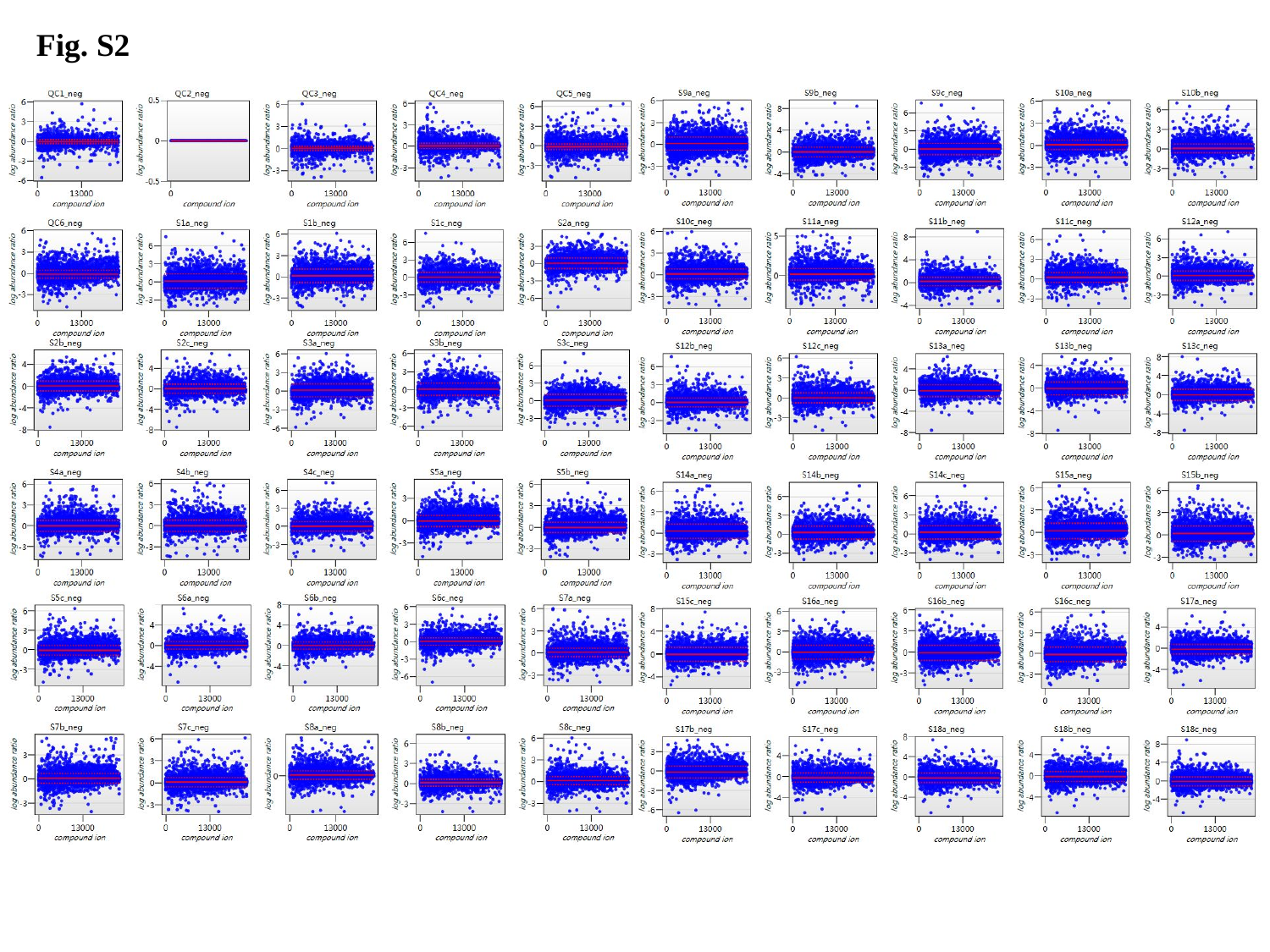

Fig. S2

Supplement: Supplementary file 3 — Additional file 3: Figure S2. The normalization graphs of RPM samples from different geographical origins. [file 13020_2017_155_MOESM3_ESM.pptx]
